# Stretching the imagination beyond muscle spindles – stretch‐sensitive mechanisms in arthropods

**DOI:** 10.1111/joa.12329

**Published:** 2015-06-16

**Authors:** Thomas J. Suslak, Andrew P. Jarman

**Affiliations:** ^1^ Doctoral Training Centre in Neuroinformatics and Neural ComputationUniversity of EdinburghEdinburghUK; ^2^ Centre for Integrative PhysiologyUniversity of EdinburghEdinburghUK

**Keywords:** invertebrate, mechanotransduction, proprioception, sensory, stretch receptor

## Abstract

Much attention has been given to mammalian muscle spindles and their role in stretch‐mediated muscle proprioception. Recent studies, particularly, have sought to determine the molecular mediators of stretch‐evoked mechanotransduction, which these endings rely upon for functionality. Nonetheless, much about these endings remains unknown. Opportunities may be presented from consideration of extensive parallel research in stretch receptor mechanisms in arthropods. Such systems may provide a useful source of additional data and powerful tools for dissecting the complex systems of stretch transduction apparatus. At the least, such systems provide tractable exemplars of how organisms solve the problem of converting stretch stimuli to electrical output. Potentially, they may even provide molecular mechanisms and candidate molecular mediators of direct relevance to mammalian muscle spindles. Here we provide a brief overview of research on arthropod stretch receptors.

## Introduction

To control posture and movement, animals with muscles sense muscle contraction and stretching via dedicated sensory organs, either directly or indirectly. Such muscle stretch receptor organs (SROs) are exemplified by the well‐known mammalian muscle spindle (Bewick et al. 2005; Simon et al. 2010; Bewick & Banks, 2015). Invertebrate animals have a similar requirement for sensing muscle stretch, and such organisms represent a huge pool of biological diversity to be explored for mechanisms. As in other areas of neuroscience and physiology, invertebrate mechanisms provide a parallel route for study. Specifically, understanding the anatomy and physiology of stretch sensory organs in arthropods might be helpful for our appreciation of the mammalian muscle spindle.

## Distribution and anatomy of stretch receptors in arthropods

The musculoskeletal systems of arthropods, particularly various crustaceans and insects, have a long history of study. SROs associated with muscles were first putatively identified anatomically in crustaceans by Alexandrowicz (1951). Between 1951 and 1956, extensive analyses of ‘muscle receptor organs’ (MROs) were carried out in various crustacean species, rigorously characterising what turned out to be the stereotypical morphology of these putative receptors, being characteristically bipolar in shape with the two presumed sensory terminals oriented in parallel to the direction of the major longitudinal skeletal muscle fibres (Alexandrowicz, 1951, 1952, 1956).

Subsequent studies in insects revealed similar simple putative stretch receptors (Finlayson & Loewenstein, 1958). These generally consist of single type II (i.e. non‐ciliated) sensory neurons associated with strands of muscle or connective tissue (Bräuning et al. 1986). Early studies identified a particular receptor that is generally termed the dorsal longitudinal stretch receptor or SRO. This SRO appears to be present in most thoracic and abdominal segments in larvae or adults of all insect orders investigated (Finlayson & Loewenstein, 1958; Osborne & Finlayson, 1962). The SRO consists of a sensory neuron with two sensory processes orientated longitudinally across the segment (Finlayson, 1976). In fact, the presence of these bipolar dendritic neurons was first documented in the 18th century by the Dutch lawyer and amateur naturalist, Pierre Lyonet (Encyclopedia Brittanica, 2014), in superbly detailed drawings of the peripheral nervous system of the goat moth caterpillar, *Cossus cossus* L. (Lyonet, 1760). His drawings clearly show the presence in each segment of a pair of neurons with bipolar sensory processes, one on each side of the body wall, with their termini aligned across the longitudinal axis. Whilst the SRO is clearly homologous between diverse insects, there are differences in detail between the insect neurons, with bipolar dendrites, and those of crustaceans, in which more complex neuronal projections appear to insert into a longitudinal muscle capsule structure, and it is therefore unclear as to whether the insect SRO and crustacean MRO are completely homologous. Nevertheless, the presence of receptor termini arranged parallel to the main axis of stretch hints at a common function, and is also notably reminiscent of the arrangement of spindles within mammalian muscles.

**Figure 1:**
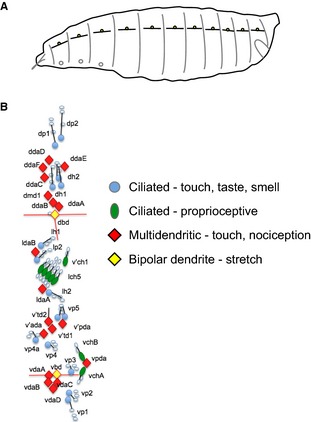
Sensory neurons of the *Drosophila* larva. (A) Schematic of the larva indicating the approximate segmental locations of the chain of dorsal bipolar dendrite (dbd) neurons, with their dendrites lying in the longitudinal axis. (B) Schematic of the sensory neurons found on each side of a single abdominal segment. Naming convention is after Bodmer & Jan (1987). Neuron cell bodies are colour‐coded according to morphology and function. Of the neurons shown, the dbd neuron (filled in yellow) is the putative stretch receptor. Another putative bipolar dendrite neuron is found ventrally (vbd neuron), but the nature and function of this neuron is uncharacterised.

**Figure 2:**
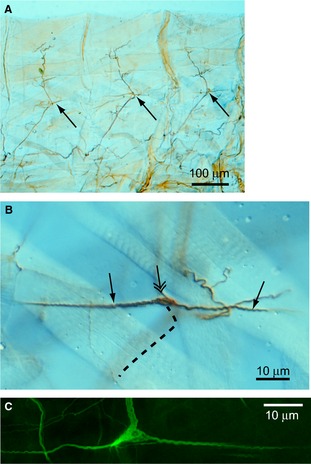
The dbd neuron of the *Drosophila* larva. (A) Light micrograph of the dorsolateral regions of three abdominal segments. The peripheral nerves are visualised by anti‐horseradish peroxidase staining. The dendrites of the dbd neurons are indicated by arrows. Some of the longitudinal muscle fibres that run in parallel are visible above the neurons. (B) Higher‐magnification light micrograph of a dbd neuron stained with anti‐horseradish peroxidase. The horizontal dendrites are arrowed and the cell soma indicated by a double arrow. Note that the dendrites often appear somewhat helical. The axon of the neuron runs ventrally but is out of focus: its approximate trajectory is indicated by the dotted line. It joins the main segmental nerve (just visible out of focus in the image). Some oblique muscle fibres are visible in the background. (C) Immunofluorescence image of dbd neuron, labelled by expression of green fluorescent protein. The large nerve visible above the cell soma is the segmental nerve that passes close to the dbd neuron.

In more recent years, these structures have been described in detail in the tobacco hornworm, *Manduca sexta* (L.), the larva of a type of hawkmoth (Tamarkin & Levine, 1996) and also in the fruit fly, *Drosophila melanogaster* Meigen (Schrader & Merritt, 2007). Studies in a range of insect orders show the dorsal longitudinal neurons to be associated along muscles. In *Manduca* the SRO consists of the bipolar neuron embedded within a thin ‘receptor muscle’ (Tamarkin & Levine, 1996). However, the equivalent neuron appears different in *Drosophila*. In surveys mapping the larval sensory nervous system of *Drosophila*, the equivalent cell to the SRO neuron has been termed the dorsal bipolar dendrite (dbd) neuron (Fig. 1; Bodmer & Jan, 1987). This cell is in a similar location to other SROs and has a similar anatomy, with dendrites lying parallel to the main longitudinal muscle fibres. However, in this case the dendrites are not located along muscles directly, but instead are physically associated with a thin connective tissue strand and the distal tips of the sensory projections are attached to epidermal cells at the segment boundaries, similarly to the longitudinal muscle insertions themselves (Figs 1 and 2; Schrader & Merritt, 2007).

Much is known of the developmental origin of sensory neurons in *Drosophila*. The dbd neuron has several developmental features that are either shared with or distinct from other sensory neurons (Lai & Orgogozo, 2004). All sensory neurons originate from individual precursor cells derived from the ectoderm by the expression of a proneural transcription factor (Jarman, 2002). Unusually, the dbd neuron precursor cell requires the proneural gene, *amos*, which is otherwise mainly responsible only for olfactory sensory neurons (Huang et al. 2000; zur Lage et al. 2003). Moreover, the dbd precursor cell divides only once to give rise to the single dbd neuron and a cell of glial nature. This division pattern is quite distinct from that of all other sensory precursor cells, although comparative analysis has suggested that the dbd division pattern, a single division to give the neuron and its ensheathing sister glial cell, may in fact represent the ancestral state for sensory precursors (Lai & Orgogozo, 2004). During insect metamorphosis, many sensory neurons are completely replaced; however, the *Drosophila* larval dbd neurons have been shown to persist into the adult (Shepherd & Smith, 1996).

## Stretch receptor function

Clearly, the SRO and related MRO are in a position to provide proprioceptive feedback during segmental muscle contraction and relaxation. In caterpillars, the SRO responds (as measured by firing rates) both tonically to slow stretch and phasically to rapid stretch (Osborne & Finlayson, 1965; Tamarkin & Levine, 1996; Simon & Trimmer, 2009). Moreover, SRO stimulation results in muscle stretch reflexes (Tamarkin & Levine, 1996). In species where these stretch receptors are associated with a sensory ‘receptor muscle’, it appears that this allows modulation of stretch receptor response by efferent feedback – a situation somewhat reminiscent of the fusiform fibre workings of mammalian muscle spindles. In *Manduca*, the SRO neuron sends an axon to the central nervous system that makes direct synaptic connections with motor neurons (Tamarkin & Levine, 1996). It appears that motor neurons innervating muscles close to the receptor receive excitatory input, whereas muscles on the opposite side of the body are inhibited (Tamarkin & Levine, 1996).

Despite the functional evidence for the *Manduca* caterpillar SRO as a proprioceptor, a surprising observation is that surgical ablation of the SRO in one or several segments did not appear to result in a change in various movement behaviours (Simon & Trimmer, 2009). A role as an error detector is possible, or even some kind of developmental role, such as regulating larval moulting through responding to body size. The functional significance of this stretch receptor clearly requires more attention. In this context, the experimental tools available in *Drosophila* may suggest that this is one way forward. For example, genetic approaches could be used to ablate or silence all the dbd neurons in an individual, either developmentally or functionally, rather than surgically.

## Physiology of arthropod stretch receptors

Electrophysiological studies have confirmed the stretch receptor action of these organs, and have often drawn parallels between their characteristics and those of vertebrate muscle spindles. Eyzaguirre & Kuffler (1955) demonstrated that a micrometre stretch stimulus applied to an intact nerve–muscle preparation from the crayfish *Procambarus alleni* (Faxon), *Orconectes virilis* (Hagen) and *Homarus americanus* Milne‐Edwards evoked excitability of the afferent nerve. Notably, this study cites the methods of Katz, investigating stretch‐evoked excitability of frog muscle spindles, as inspiration (Katz, 1950a,b). In particular, Eyzaguirre & Kuffler make special mention, on a couple of occasions, that their recordings bear similarities to the observations of Katz.

Later examinations of the electrophysiological responses of stretch receptors have attempted to define the nature of the receptor potential generated in response to stretch. Work on the MRO of the crayfish *Astacus astacus* L. and *Pacifastacus leniusculus* (Dana) demonstrated the presence in these endings of stretch‐activated tetrodotoxin‐ (TTX) and tetra‐ethyl ammonium‐ (TEA) sensitive currents (Ottoson & Swerup, 1985a,b), suggesting the contribution of voltage‐gated Na^+^ channels and voltage‐gated K^+^ channels, respectively (Kaila et al. 1987). A further Na^+^ current component was detected that was activated by mechanical stimulation but not blocked by TTX. In addition, some Ca^2+^‐dependence of the receptor potential that could be recorded in the sensory terminals was reported (Edwards et al. 1981).

**Figure 3:**
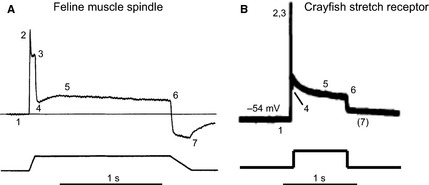
Receptor potentials of rodent muscle spindles and crayfish stretch receptors compared. (A) Receptor potential recorded from primary axon of a muscle spindle afferent in response to a stretch stimulus, shown below the trace (adapted from Hunt et al. (1978)). (1) Baseline; (2) peak of initial dynamic component; (3) peak of late dynamic; (4) post‐dynamic minimum; (5) static maximum; (6) end static level; and (7) post‐release minimum. (B) A receptor potential of crayfish SRO in response to stretch (adapted from Ottoson & Swerup, 1982). Resting membrane potential is indicated to the left of the trace. The crayfish trace has been annotated in a similar fashion to (A). In this interpretation, several features of the muscle spindle response are identifiable in the crayfish data. A notable exception is lack of post‐release minimum (7). Other differences may be the result of the step‐change stretch applied rather than a ramp stimulus.

What is particularly interesting about the receptor potentials of crayfish stretch receptors, as observed by, for example, Ottoson & Swerup (1982), is the similarity in waveform to analogous recordings made in rodent muscle spindles (Hunt et al. 1978). That is, recordings of receptor potentials in both crayfish stretch receptors and rodent muscle spindles exhibit an ‘on’ response to stretch, characterised by a large depolarisation response to dynamic stretch, an adaptive repolarisation to a ‘hold potential’ (persistent depolarised state) that is proportional to the amplitude of a static stretch stimulus, and an ‘off’ response as stimuli are terminated, resulting in a return to resting membrane potential (Fig. 3). In the muscle spindle study, intracellular sharp electrode measurements of stretch‐evoked receptor potentials, made from TTX‐poisoned axons of Ia afferents, had also reported the ionic dependencies observed in the MRO: a strong TTX‐insensitive mechanosensory Na^+^ current, a smaller (~20%) contribution from Ca^2+^, and a TEA‐dependent K^+^ current (Hunt et al. 1978). The parallels are striking and, indeed, strongly point to the potential for some degree of mechanistic correlation between the two systems.

More recently, an analysis of the ionic dependencies of the dbd neuron of *Drosophila* has added further detail to support the anatomical evidence for its relationship to other arthropod stretch receptors (Nair et al. 2010). This study identified, via whole‐cell voltage‐clamp recordings, distinct 4‐aminopyridine‐sensitive and TEA‐sensitive K^+^ currents, and a Na^+^ current blocked by TTX, as well as a voltage‐activated Ca^2+^‐current. Again, these distinctly resembled earlier recordings from stretch receptors of other species (Edwards et al. 1981; Ottoson & Swerup, 1982). Nair et al. (2010) concluded that their method might prove a valuable tool in finding further evidence to support the hypothesis that dbd neurons are likely stretch receptor candidates in *Drosophila*. Indeed, in our own work we have successfully adapted this protocol to confirm the existence of mechanically evoked potential responses in the dbd neuron dendrites of filleted larvae (Suslak et al. 2015).

## Mediators of mechanotransduction

The nature of the mechanical stimulation leading to mechanotransduction is not known. In some insects it is proposed that free dendritic endings are squeezed during muscle or connective tissue stretch (Osborne & Finlayson, 1965). In the *Drosophila* dbd neuron, detailed structural analysis led to the proposal that mechanical stimulation results in dendritic membrane tension or in shearing forces between the dendrite and the surrounding glial cell (Schrader & Merritt, 2007).

Despite a wealth of anatomical and physiological knowledge, little is known of the molecular mediators of mechanotransduction in arthropod stretch receptors. In one approach, the application of mathematical modelling of these receptor types opens up a useful avenue of comparative study. Edwards et al. (1981) demonstrated that the Goldman–Hodgkin–Katz equation (Hodgkin & Katz, 1949) could be utilised in crayfish stretch receptors to estimate the ionic dependence of stretch‐induced currents in the afferent ending. This method was further extended to model both the tension in the sensory endings as a Voigt element (spring and dashpot) and the role of a mechanosensory Na^+^ current in stretch‐mediated receptor depolarisation and receptor potential generation (Swerup & Rydqvist, 1996). Our own work has developed this *in silico* approach to reproduce both the stretch‐ and voltage‐mediated currents observed by Ottoson & Swerup (1982). Significantly, this biophysical model can account for the complex receptor potential observed in both arthropod and mammalian stretch receptors (Suslak et al. 2011, 2015).

In addition to this general approach, there is great potential in screening for mechanotransduction molecules using *Drosophila*, both to understand SRO function as a model proprioceptor and possibly also to inform the search for similar mediators in vertebrate muscle spindles. Not only do *Drosophila* possess a well‐described example of an arthropod stretch receptor in the dbd neuron, but the sensory projections of these neurons have been shown to be amenable to patch‐clamp electrophysiology in order to obtain direct measurements of receptor potential changes (Baines & Bate, 1998; Nair et al. 2010). Such information has thus far been difficult to obtain in vertebrate muscle spindles. Indeed, we have recently demonstrated that receptor potentials of intact dbd neurons in response to a stretch stimulus can be recorded *in situ* (Suslak et al. 2015). The receptor potential profile of the dbd neuron appears strikingly similar to that of mammalian muscle spindle shown in Fig. 3A, including the post‐stretch hyperpolarisation component (Suslak et al. 2015).

When combined with the powerful genetic toolbox available for *Drosophila*, this system has the potential to yield a wealth of data that could greatly advance our knowledge of mechanotransduction mechanisms. Indeed, several putative mechanosensory channels are known to be expressed in dbd neurons, including the *Drosophila* orthologues of Piezo and TRPA1 (Kim et al. 2010, 2012; Shen et al. 2011), and our recent work using a combination of pharmacology, genetics and electrophysiology has indicated that both channels play a role in dbd neuron receptor potential generation (Suslak et al. 2015). Interestingly, there is as yet no evidence for a role for ENaC channels in dbd neurons (Suslak et al. 2015), although they are strong candidates for involvement in mammalian muscle spindles (Simon et al. 2010). Another apparent difference with muscle spindles is that the ultrastructure of dbd neurons (Schrader & Merritt, 2007) appears to give no support for the action of an autogenic glutamate mechanism based on release of dendritic vesicles, as has been demonstrated for muscle spindles (Bewick et al. 2005; Bewick & Banks, 2015).

Currently, then, study of stretch receptors of arthropods presents a useful avenue for addressing the question of what may mediate stretch‐evoked responses in mechanoreceptors. Moving forward, the use of the model organism *D*. *melanogaster* may provide much new insight into what constitutes a mechanosensory mechanism. Such insight may provide an intriguing and useful comparison to the mammalian muscle spindle.

## References

[joa12329-bib-0001] Alexandrowicz JS (1951) Muscle receptor organs in the abdomen of *Homarus vulgaris* and *Palinurus vulgaris* . Quart J Micr Sci 92, 163–199.

[joa12329-bib-0002] Alexandrowicz JS (1952) Muscle receptor organs in the *Paguridae* . J Mar Biol Ass UK 31, 277–286.

[joa12329-bib-0003] Alexandrowicz JS (1956) Receptor elements in the muscles of *Leander serratus* . J Mar Biol Ass UK 35, 129–144.

[joa12329-bib-0004] Baines RA , Bate M (1998) Electrophysiological development of central neurons in the *Drosophila* embryo. J Neurosci 18, 4673–4683. 9614242 10.1523/JNEUROSCI.18-12-04673.1998 PMC6792699

[joa12329-bib-0005] Bewick GS , Reid B , Richardson C , et al. (2005) Autogenic modulation of mechanoreceptor excitability by glutamate release from synaptic‐like vesicles: evidence from the rat muscle spindle primary sensory ending. J Physiol 562, 381–394. 15528245 10.1113/jphysiol.2004.074799 PMC1665510

[joa12329-bib-0006] Bewick GS , Banks RW (2015) Mechanotransduction in the muscle spindle. Eur J Physiol 1, 175–190. 10.1007/s00424-014-1536-9 PMC4281366 24888691

[joa12329-bib-0007] Bräuning P , Cahill MA , Hustert R (1986) The coxo‐trochanteral muscle receptor organ of locusts. Dendritic tubular bodies in a non‐ciliated insect mechanoreceptive neuron. Cell Tissue Res 243, 517–524.

[joa12329-bib-0008] Bodmer R , Jan YN (1987) Morphological differentiation of the embryonic peripheral neurons in *Drosophila* . Roux Arch Dev Biol 196, 69–77. 10.1007/BF00402027 28305460

[joa12329-bib-0009] Edwards C , Ottoson D , Rydqvist B , et al. (1981) The permeability of the transducer membrane of the crayfish stretch receptor to calcium and other divalent cations. Neuroscience 8, 1455–1460. 10.1016/0306-4522(81)90200-1 6267508

[joa12329-bib-0010] Encyclopedia Brittanica (2014) http://www.britannica.com/EBchecked/topic/352981/Pierre-Lyonnet. Accessed 5 December 2014.

[joa12329-bib-0011] Eyzaguirre C , Kuffler SW (1955) Processes of excitation in the dendrites and in the soma of single isolated sensory nerve cells of the lobster and crayfish. J Gen Physiol 39, 87–119. 13252237 10.1085/jgp.39.1.87 PMC2147518

[joa12329-bib-0501] Finlayson LH (1976) Abdominal and thoracic receptors in insects, centipedes and scorpions In: Structure and Function of Proprioceptors in the Invertebrates (ed. Mill PJ), pp. 153–211. London: Chapman & Hall.

[joa12329-bib-0012] Finlayson LH , Loewenstein O (1958) The structure and function of abdominal stretch receptors in insects. Proc R Soc Lond B Biol Sci 148, 433–449. 13542636 10.1098/rspb.1958.0037

[joa12329-bib-0013] Hodgkin AL , Katz B (1949) The effect of sodium ions on the electrical activity of the giant axon of the squid. J Physiol 108, 37–77. 18128147 10.1113/jphysiol.1949.sp004310 PMC1392331

[joa12329-bib-0014] Huang ML , Hsu CH , Chien CT (2000) The proneural gene *amos* promotes multiple dendritic neuron formation in the *Drosophila* peripheral nervous system. Neuron 25, 57–67. 10707972 10.1016/s0896-6273(00)80871-5

[joa12329-bib-0015] Hunt CC , Wilkinson RS , Fukami Y (1978) Ionic basis of the receptor potential in primary endings of mammalian muscle spindles. J Gen Physiol 71, 683–698. 149839 10.1085/jgp.71.6.683 PMC2215112

[joa12329-bib-0016] Jarman AP (2002) Studies of mechanosensation using the fly. Hum Mol Genet 11, 1215–1218. 12015281 10.1093/hmg/11.10.1215

[joa12329-bib-0017] Kaila K , Rydqvist B , Swerup C , et al. (1987) Stimulation‐induced changes in the intracellular sodium activity of the crayfish stretch receptor. Neurosci Lett 74, 53–57. 2436106 10.1016/0304-3940(87)90050-4

[joa12329-bib-0018] Katz B (1950a) Action potentials from a sensory nerve ending. J Physiol 111, 248. 14795438 10.1113/jphysiol.1950.sp004478 PMC1392829

[joa12329-bib-0019] Katz B (1950b) Depolarization of sensory terminals and the initiation of impulses in the muscle spindle. J Physiol 111, 261. 14795439 10.1113/jphysiol.1950.sp004479 PMC1392822

[joa12329-bib-0020] Kim SE , Coste B , Chadha A , et al. (2012) The role of *Drosophila* Piezo in mechanical nociception. Nature 483, 209–213. 22343891 10.1038/nature10801 PMC3297676

[joa12329-bib-0021] Kim SH , Lee Y , Akitakea B , et al. (2010) *Drosophila* TRPA1 channel mediates chemical avoidance in gustatory receptor neurons. PNAS 107, 8440–8445. 20404155 10.1073/pnas.1001425107 PMC2889570

[joa12329-bib-0022] Lai EC , Orgogozo V (2004) A hidden program in *Drosophila* peripheral neurogenesis revealed: fundamental principles underlying sensory organ diversity. Dev Biol 269, 1–17. 15081353 10.1016/j.ydbio.2004.01.032

[joa12329-bib-0023] Lyonet P (1760) Traité anatomique de la chenille, qui ronge le bois de saule. Haye, Amsterdam.

[joa12329-bib-0024] Nair A , Bate M , Pulver SR (2010) Characterization of voltage‐gated ionic currents in a peripheral sensory neuron in larval *Drosophila* . BMC Res Notes 3, 154–161. 20525165 10.1186/1756-0500-3-154 PMC2893198

[joa12329-bib-0025] Osborne MP , Finlayson LH (1962) The structure and topography of stretch receptors in representatives of seven orders of insects. Quart J Microsc Sci 103, 227–242.

[joa12329-bib-0026] Osborne MP , Finlayson LH (1965) An electron microscope study of the stretch receptor of *Antheraea pernyi* (Lepidoptera, Saturniidae). J Insect Physiol 11, 703–710. 5827532 10.1016/0022-1910(65)90152-6

[joa12329-bib-0027] Ottoson D , Swerup C (1982) Studies on the role of calcium in adaptation of the crustacean stretch receptor. Effects of intracellular injection of calcium. EGTA and TEA. Brain Res 244, 337–341. 6288188 10.1016/0006-8993(82)90093-2

[joa12329-bib-0028] Ottoson D , Swerup C (1985a) Ionic dependence of early adaptation in the crustacean stretch receptor. Brain Res 336, 1–8. 4005570 10.1016/0006-8993(85)90409-3

[joa12329-bib-0029] Ottoson D , Swerup C (1985b) Effects of intracellular TEA injection on early adaptation of crustacean stretch receptor. Brain Res 336, 9–17. 4005580 10.1016/0006-8993(85)90410-x

[joa12329-bib-0030] Schrader S , Merritt DJ (2007) Dorsal longitudinal stretch receptor of *Drosophila melanogaster* larva – fine structure and maturation. Arthropod Struct Dev 36, 157–169. 18089096 10.1016/j.asd.2006.08.014

[joa12329-bib-0031] Shen WL , Kwon Y , Adegbola AA , et al. (2011) Function of rhodopsin in temperature discrimination in *Drosophila* . Science 331, 1333–1336. 21393546 10.1126/science.1198904

[joa12329-bib-0032] Shepherd D , Smith SA (1996) Central projections of persistent larval sensory neurons prefigure adult sensory pathways in the CNS of *Drosophila* . Development 122, 2375–2384. 8756283 10.1242/dev.122.8.2375

[joa12329-bib-0033] Simon A , Shenton F , Hunter I , et al. (2010) Amiloride‐sensitive channels are a major contributor to mechanotransduction in mammalian muscle spindles. J Physiol 588, 171–185. 19917568 10.1113/jphysiol.2009.182683 PMC2821557

[joa12329-bib-0034] Simon MA , Trimmer BA (2009) Movement encoding by a stretch receptor in the soft‐bodied caterpillar, *Manduca sexta* . J Exp Biol 212, 1021–1031. 19282499 10.1242/jeb.023507

[joa12329-bib-0035] Suslak TJ , Armstrong JD , Jarman AP (2011) A general mathematical model of transduction events in mechano‐sensory stretch receptors. Network 22, 133–142. 22149673 10.3109/0954898X.2011.638967

[joa12329-bib-0036] Suslak TJ , Watson S , Thompson KJ , et al. (2015) Piezo is essential for amiloride‐sensitive stretch‐activated mechanotransduction in larval *Drosophila* dorsal bipolar dendritic sensory neurons. PLoS ONE in press. 10.1371/journal.pone.0130969 PMC4506125 26186008

[joa12329-bib-0037] Swerup C , Rydqvist B (1996) A mathematical model of the crustacean stretch receptor neuron. Biomechanics of the receptor muscle, mechanosensitive ion channels, and macrotransducer properties. J Neurophysiol 76, 2211–2220. 8899596 10.1152/jn.1996.76.4.2211

[joa12329-bib-0038] Tamarkin DA , Levine RB (1996) Synaptic interactions between a muscle‐associated proprioceptor and body wall muscle motor neurons in larval and adult *Manduca sexta* . J Neurophysiol 76, 1597–1610. 8890279 10.1152/jn.1996.76.3.1597

[joa12329-bib-0039] zur Lage PI , Prentice DRA , Holohan EI , et al. (2003) The *Drosophila* proneural gene *amos* promotes olfactory sensillum formation and suppresses bristle formation. Development 130, 4683–4693. 12925594 10.1242/dev.00680

